# Impact of the Russia-Ukraine Conflict on International Staple Agrifood Trade Networks

**DOI:** 10.3390/foods13132134

**Published:** 2024-07-04

**Authors:** Yin-Ting Zhang, Mu-Yao Li, Wei-Xing Zhou

**Affiliations:** 1 School of Business, East China University of Science and Technology, Shanghai 200237, China; ytzhang@mail.ecust.edu.cn (Y.-T.Z.); myli@mail.ecust.edu.cn (M.-Y.L.); 2 Research Center for Econophysics, East China University of Science and Technology, Shanghai 200237, China; 3 Department of Mathematics, East China University of Science and Technology, Shanghai 200237, China

**Keywords:** food security, international staple agrifood trade networks, network structure, Russia-Ukraine conflict

## Abstract

The Russia-Ukraine conflict is a growing concern worldwide and poses serious threats to regional and global food security. Using monthly trade data for maize, rice, and wheat from 2016/1 to 2023/12, this paper constructs three international crop trade networks and an aggregate international food trade network. We aim to examine the structural changes following the occurrence of the Russia-Ukraine conflict. We find significant shifts in the number of edges, average in-degree, density, and efficiency in the third quarter of 2022, particularly in the international wheat trade network. Additionally, we have shown that political reasons have caused more pronounced changes in the trade connections between the economies of the North Atlantic Treaty Organization and Russia than with Ukraine. This paper could provide insights into the negative impact of geopolitical conflicts on the global food system and encourage a series of effective strategies to mitigate the negative impact of the conflict on global food trade.

## 1. Introduction

The Russia-Ukraine conflict is a growing concern worldwide [1,2]. It has had significant ramifications not only on political and security fronts but also on various socio-economic aspects, including economic development [3,4], regional environment [5,6], international trade [7,8], food supply chains [9], and food security [10,11,12]. Since Russia and Ukraine have emerged as key players in the global agricultural and energy markets over the past few years [13], the conflict poses serious global and regional food and energy security challenges [14,15].

According to UN Comtrade (Accessed on 15 July 2023, see https://comtradeplus.un.org/) and FAO (Accessed on 15 July 2023, see https://www.fao.org/faostat/en/), Russia and Ukraine together accounted for significant shares of global wheat, maize, and sunflower oil exports, representing approximately 34 percent, 17 percent, and 73 percent, respectively. Additionally, they held substantial market shares in the global barley and maize trade, contributing around 27 percent and 17 percent, respectively, [16]. These exports play a crucial role in global consumption and diets, providing approximately 12 percent of the total calories traded worldwide. Furthermore, Russia holds a significant position as a major exporter of nitrogen and potash fertilizers, contributing to approximately 15 percent of global trade in nitrogenous fertilizers. Collectively, Russia and Belarus account for approximately 33 percent of global potash fertilizer exports [17].

Food trade is crucial to global food systems [18,19]. Economies and trade relationships create global food trade networks [20,21]. Trade provides a way to bridge the gap between regions with surplus food production and those facing deficits, thereby contributing to global food security goals. However, climate change [22,23], geopolitical conflict [24,25], pandemics [19,26], and the financial crisis [27] will all conspire to put unprecedented stress on the global food trade system. When economies that are main food producers or exporters experience a reduction in food production [28] due to extreme events or the implementation of trade restrictions, it will result in shortages, price spikes, and increased vulnerability to hunger and malnutrition. Therefore, the strong presence of Russia and Ukraine in agricultural sectors underscores their importance in global food trade and food security.

Econometric equilibrium models, such as Computable General Equilibrium (CGE) models, are frequently employed to analyze the impact of external shocks on trade and food security. These models simulate how economies respond to changes in policy, technology, and other external factors by capturing interactions between different sectors and regions. Previous studies have effectively used CGE models to explore the effects of various global disruptions. Some studies utilized a CGE model to examine the economic repercussions of climate change on global agriculture, finding significant variations in regional impacts [29,30,31]. Similarly, Chalise et al. (2017) applied a CGE model to assess the implications of potential yield improvements and trade liberalization on global food security, demonstrating the critical role of productivity gains in mitigating food insecurity [32,33]. In the context of the Russia-Ukraine conflict, CGE models have been used to quantify the impacts of the Russia-Ukraine conflict on global food security, agricultural production, trade dynamics, and greenhouse gas emissions. For example, a study employs a CGE model to examine the impact of international oil price fluctuations and carbon tax policies on China’s energy-economic-environment system [34]. Liu et al. employ the CGE model to assess the environmental and economic impacts of embargoing Russian fossil fuel imports on the European Union [35].

Recent studies have dedicated attention to examining the potential impacts of the Russia-Ukraine conflict on food security from various perspectives. One branch of this literature analyzes the effects of the conflict on food yields [36]. The scarcity of labor, suspension of transportation, and disturbances to the supply of chemical fertilizers, as well as pest and disease controls, could have a profound impact on wheat cultivation in Ukraine. Additionally, several studies have explored the influence of the conflict on global food prices [37,38], trade [39,40,41,42,43,44], and food supply chain [9,10,45]. These studies have found that the conflict would cause sharp increases in food prices, a decline in trade, and acute food insecurity, particularly for nations that rely significantly on grain imports from Russia and Ukraine [46,47]. Furthermore, researchers have investigated how shocks generated by the conflict propagate through the global food trade network [48].

Some of these studies have utilized historical data prior to 2023 [8] or employed algorithms and models to simulate the impact of the Russia-Ukraine conflict on the food system [46,49,50]. However, international food trade networks evolve [51] and there is a lack of research that has examined the effects of the conflict using post-conflict food trade data. In this paper, we leverage monthly trade data for maize, rice, and wheat from January 2016 to December 2023, obtained from the UN Comtrade Database, to construct three distinct international crop trade networks and an aggregated international food trade network. By comparing the changes in network structure and properties before and after the Russia-Ukraine conflict, we aim to analyze its influence on the global food trade network.

Due to the inherent delays in data collection and updates, most economies, including Russia and Ukraine, have not provided food trade data beyond 2023. Only a subset of European economies and a small number of economies in North and South America have reported timely and reliable trade data. To address this limitation, we fill in the missing data using bilateral trade data provided by the reporting economies. This approach enables us to capture the trade relationships between Russia, Ukraine, and select economies. Although we cannot construct a comprehensive global food trade network, our study still provides valuable insights into parts of global food trade networks, thereby filling a research gap in analyzing the actual impact of the Russia-Ukraine conflict using up-to-date data. Based on network construction and formulation of topological metrics, we highlight the effects of the Russia-Ukraine conflict on the three international crop trade networks, the aggregate international food trade network, and economies.

## 2. Data and Method

### 2.1. Data Description

We consider three crops (maize, rice, and wheat) that meet the main calorie needs of the world’s population. Our research incorporates monthly import and export data for these crops, spanning from January 2016 to December 2023, sourced from the UN Comtrade database. Due to delayed updates in data reporting, numerous economies have not yet provided monthly trade data for these crops since 2023. To mitigate this data gap, we have adopted a strategy that leverages information from trade partners to supplement the missing data. When a particular economy, such as China, fails to disclose its crop import data for a given month (e.g., April 2022), we rely on data reported by other countries, such as Brazil, who have shared their crop export data with China during the same period. By extrapolating from Brazil’s data, we estimate China’s crop import volume for that specific month. Likewise, in instances where Russia has not reported any crop trade data since April 2022, we utilize import and export data provided by its trade partners to fill in the gaps and obtain a comprehensive picture of Russia’s crop trade activity during that period.

We establish trade relationships between reporting economies and their trading partners. Because of big monthly swings, we perform quarterly analysis of the international crop trade networks rather than monthly analysis, thereby maximizing coverage of the economies included in our study. Since many economies do not report trade data for 2024 and before 2016, we only consider international food trade data between 2016 and 2023. Following data clean-up, we focus on those economies that constantly provided data during the period from January 2016 to December 2023, which include 42 reporting economies for maize, 43 for rice, and 25 for wheat (see Table 1). To compare and aggregate different crop trade data, we ultimately use three types of crop trade data provide by 24 reporting economies (see the reporting economies for crops shown in Table 1). Our dataset contains crop trade flows for the selected economies only. As a result, we are unable to investigate the Russia-Ukraine conflict’s impact on some economies, which would underestimate the conflict’s impact on the global crop trade system. Nevertheless, we could still establish international food trade networks based on the available data, allowing us to assess the conflict’s effects on some economies’ crop trade.

### 2.2. Network Construction

Since monthly trade data possess significant fluctuations, we restrict ourselves to the case of quarterly trade evolution. To eliminate the effects of crop prices and quality, we use food conversion data provided by the Food and Agriculture Organization of the United Nations (FAO) to convert the trade volumes into trade calories. Based on monthly trade data provided by the 24 economies selected, we derive the quarterly trade matrix $W^{crop}\left(t\right)$ for each crop in each quarter *t* (crop= maize, rice, and wheat, t= 2016/Q1, 2016/Q2, ⋯, 2023/Q4), where $w_{ij}^{crop}\left(t\right)$ denotes the caloric trade volume of crop exported from economy *i* to economy *j*. For each crop in each quarter from January 2016 to December 2022, we construct a time-varying international crop trade network (iCTN), $G^{crop}\left(t\right)=\left(V^{crop}\left(t\right),W^{crop}\left(t\right)\right)$, where $V^{crop}\left(t\right)$ denotes the set of network nodes, which includes reporting economies and their trading partners. Thus, we obtain three iCTNs: the international maize trade network (iMTN), the international rice trade network (iRTN), and the international wheat trade network (iWTN). At the same time, we construct the aggregate international food trade network (iFTN), $G\left(t\right)=\left(V(t),W(t)\right)$ by aggregating the three crops.

**Figure 1:**
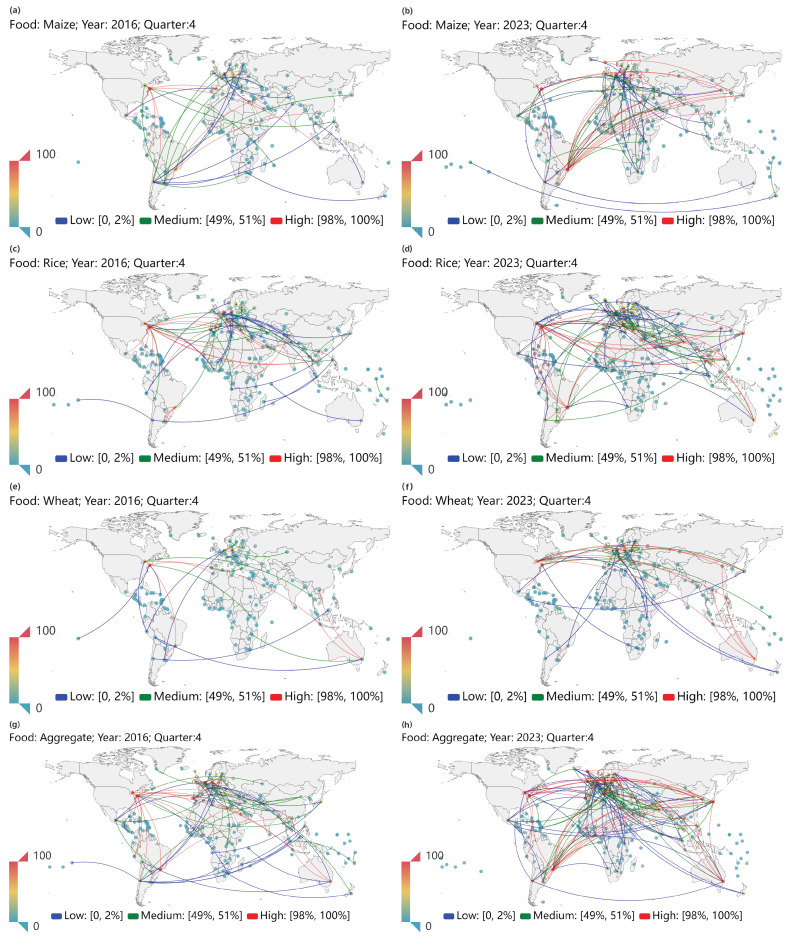
Three international crop trade networks and the international food trade network in 2016/Q4 (left column) and 2023/Q4 (right column). The rows from top to bottom, respectively, describe maize (**a**,**b**), rice (**c**,**d**), wheat (**e**,**f**), and aggregate (**g**,**h**) crops. To enhance clarity, we show only links with trade volumes ranking in the top 2%, medium 1%, and bottom 2% for each crop.

We compare the international crop trade networks and the aggregate international food trade network in different years but in the same quarter in order to exclude the seasonal impacts. Figure 1 displays the three international crop trade networks for maize, rice, and wheat as well as the aggregate international food trade network in the fourth quarter of 2016 and 2023. The rows show trade flows for each crop and food (aggregate crops) in the fourth quarter of 2016 and the fourth quarter of 2023, respectively. To enhance clarity, we show only links with trade volumes ranking in the top 2%, medium 1%, and bottom 2% for each crop. We find that the aggregate international food trade network in the fourth quarter of 2023 has more links with high trade volumes with respect to the fourth quarter of 2016 (see Figure 1g,h). It indicates that new trade relationships are formed [52]. Figure 1c,d show that the structure of the international rice trade network covering selected economies has remained relatively stable. Further analysis based on topological indicators is required.

### 2.3. Node Attributes

#### 2.3.1. Node Degree

The node degrees present the number of trade partners of economies. In a directed network, we define both in-degree and out-degree of a node to count incoming links and outgoing links, respectively. The in-degree of a node is defined as follows:

(1) $$k_{i}^{\mathrm{in}}=\sum_{j\in V-\{i\}}I_{E}\left(e_{ji}\right)=\sum_{j=1}^{N_{V}}I_{E}\left(e_{ji}\right),$$

where $I_{E}\left(e_{ji}\right)$ is the indicator function:

(2) $$I_{E}\left(e_{ji}\right)=\left\{\begin{array}{c} 1,\;\mathrm{if}\;\;e_{ji}\in E\\ 0,\;\mathrm{if}\;\;e_{ji}\notin E \end{array}\right.$$

The out-degree of a node is defined as follows:

(3) $$k_{i}^{\mathrm{out}}=\sum_{j\in V-\{i\}}I_{E}\left(e_{ij}\right)=\sum_{j=1}^{N_{V}}I_{E}\left(e_{ij}\right).$$

#### 2.3.2. Node Strength

Since the networks are weighted, we quantifynode strengths, including in-strength $s_{i}^{\mathrm{in}}$ and out-strength $s_{i}^{\mathrm{out}}$, which are defined as follows:

(4) $$s_{i}^{\mathrm{in}}=\sum_{j\in V-\{i\}}w_{ji}=\sum_{j=1}^{N_{V}}w_{ji},$$

(5) $$s_{i}^{\mathrm{out}}=\sum_{j\in V-\{i\}}w_{ij}=\sum_{i=1}^{N_{V}}w_{ij},$$

where $w_{jj}=0$ by definition.

#### 2.3.3. PageRank

The PageRank algorithm was devised in 1997 to rank web pages in the Google search engine, and then it was used to measure the importance of nodes in a directed network in many fields. The PageRank computation proceeds iteratively over and over again to estimate the significance of a node [53]. Here, we omit the calculation process and apply PageRank to measure the influence of an economy in the international crop trade networks.

### 2.4. Network Metrics

#### 2.4.1. Average Node Degree

To show the average trade partners of all economies in the international crop trade networks and the aggregate international food trade network, we calculate the average node degree,

(6) $$\langle k^{\mathrm{in}}\rangle =\langle k^{\mathrm{out}}\rangle =\frac{N_{E}}{N_{V}}$$

and

(7) $${\langle k\rangle }_{V}=\langle k^{\mathrm{in}}+k^{\mathrm{out}}\rangle =\frac{2N_{E}}{N_{V}}.$$

#### 2.4.2. Average Node Strength

The average in-strength of nodes (i∈V) is expressed as follows:

(8) $${\langle s^{\mathrm{in}}\rangle }_{V}=\frac{1}{N_{V}}\sum_{j=1}^{N_{V}}s_{j}^{\mathrm{in}}=\frac{1}{N_{V}}\sum_{i=1}^{N_{V}}\sum_{j=1}^{N_{V}}{\left(w_{ij}\right)}^{1}=\frac{W}{N_{V}},$$

Similarly, the average out-strength of nodes is expressed as follows:

(9) $${\langle s^{\mathrm{out}}\rangle }_{V}=\frac{1}{N_{V}}\sum_{i=1}^{N_{V}}s_{i}^{\mathrm{out}}=\frac{1}{N_{V}}\sum_{i=1}^{N_{V}}\sum_{j=1}^{N_{V}}{\left(w_{ij}\right)}^{1}=\frac{W}{N_{V}}.$$

Therefore, we have

(10) $${\langle s^{\mathrm{in}}\rangle }_{V}={\langle s^{\mathrm{out}}\rangle }_{V}=\frac{W}{N_{V}}$$

and

(11) $${\langle s\rangle }_{V}={\langle s^{\mathrm{in}}+s^{\mathrm{out}}\rangle }_{V}=\frac{2W}{N_{V}}.$$

#### 2.4.3. Network Density

We use density to describe how connected nodes are in the network, which is defined as the portion of the potential links that are actual links, as follows:

(12) $$\rho =\frac{N_{E}}{N_{V}\left(N_{V}-1\right)},$$

#### 2.4.4. Link Reciprocity

Link reciprocity plays a pivotal role in shaping directed networks and is crucial for comprehending the observed network topology [54]. In conventional terms, the reciprocity of a node *i* is defined as the ratio of the number of reciprocal links $k_{i}^{R}$ to the total number of links $k_{i}$, associated with node *i* [55]:

(13) $$R_{i}=\frac{♯\left(\left\{j:e_{ij}\in E\;\&\;e_{ji}\in E\right\}\right)}{♯\left(\left\{j:e_{ij}\in E\;\mathrm{or}\;e_{ji}\in E\right\}\right)}=\frac{k_{i}^{R}}{k_{i}},$$

where

(14) $$k_{i}^{R}=♯\left(\left\{j:e_{ij}\in E\;\&\;e_{ji}\in E\right\}\right)=\sum_{j\neq i}{\left(w_{ij}w_{ji}\right)}^{0}$$

is the number of reciprocal links node *i* has. In Equation (14), we pose $0^{0}=0$. The investigation of link reciprocity provides valuable insights into the underlying dynamics and structure of complex networks, contributing to a more profound understanding of their behavior.

#### 2.4.5. Network Efficiency

Network efficiency is calculated as the average reciprocal of the shortest path lengths between pairs of nodes within a network [56,57]. Here, we calculate the efficiency as follows:

(15) $$E=\frac{1}{N_{V}\left(N_{V}-1\right)}\sum_{i\neq j}\frac{1}{d_{ij}},$$

where $d_{ij}$ is the shortest path between node *i* and node *j*. In the international crop trade networks, efficiency serves as a measure of how effectively crops are transported.

#### 2.4.6. Natural Connectivity

Natural connectivity offers a valuable approach for studying network resilience, avoiding complex computations and instead relying on the inherent structure of the network. Its primary application lies in quantifying the redundancy of alternative pathways within the network. This is achieved by computing a weighted sum of closed walks of different lengths [58]. Mathematically, the initial natural connectivity can be defined as follows:

(16) $$\begin{array}{r} NC=\ln\left(\frac{SC}{N_{V}}\right)=\ln\left(\frac{\sum_{i=1}^{N_{V}}e^{\lambda_{i}}}{N_{V}}\right), \end{array}$$

where

(17) $$\begin{array}{r} SC=\sum_{l=0}^{\infty }\frac{\mu_{l}}{l!}=\sum_{i=0}^{N_{V}}\sum_{l=0}^{\infty }\frac{\lambda_{i}^{l}}{l!}=\sum_{i=1}^{N_{V}}e^{\lambda_{i}}, \end{array}$$

where $\mu_{l}$ is the number of closed walks of length *l*, SC is the initial weighted sum of the numbers of closed walks [59], and λ is the eigenvalue of the adjacency matrix *A*.

### 2.5. Percentage Change in Metric

To better understand the structural changes in the international crop trade networks before and after the Russia-Ukraine conflict, we calculate percentage change r(t) to compare the network’s structure between two consecutive quarters spanning the period from 2016 to 2023,

(18) $$r\left(t\right)=\frac{x(t)-x(t-1)}{x(t-1)}$$

where x(t) means the value of the topological metric at time *t*. We focus on topological metrics that have undergone notable changes following the Russia-Ukraine conflict.

## 3. Results

Russia and Ukraine are major global producers and exporters of crops [36], with their crops such as maize and wheat holding significant positions in the global food trade [60]. The ongoing conflict between Russia and Ukraine is likely to exacerbate global food supply shortages, leading to a rise in food prices and endangering the food security. In this study, we analyze the evolution of the structural characteristics of three international crop trade networks and the aggregate international food trade network from January 2016 to December 2023. To better understand the impact of the Russia-Ukraine conflict, We focus on two time points before and after the outbreak of the Russia-Ukraine conflict (the second, third and forth quarters of 2021 and 2023, respectively).

### 3.1. Impact on the International Crop Trade Networks

**Figure 2:**
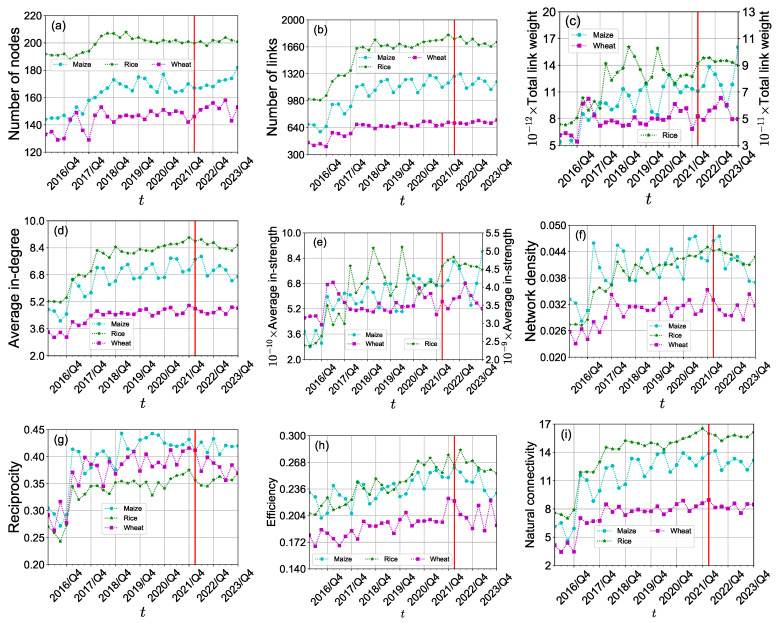
(**a**–**i**) Quarterly evolution of the structure of the three international crop trade networks from 2016/Q1 to 2023/Q4. The vertical red line corresponds to 24 February 2022 when the Russia-Ukraine conflict began.

We present the evolution of three international crop trade networks over the period from the first quarter of 2016 to the fourth quarter of 2023, highlighting various network properties such as the number of nodes, edges, total edge weight, average degree, average strength, density, clustering coefficient, efficiency, and natural connectivity, as shown in Figure 2. We find that the structure of the three international crop trade networks has significant seasonal fluctuations from 2016 to 2022 but has an increasing trend, especially after 2016. Figure 2a,b,d shows that the numbers of nodes and edges do not have a significant change. It indicates that the trade relationships between these 24 economies in the international crop trade networks are stable. It is consistent with the overall trend of the total link weights and average in-strength (see Figure 2c,e). However, the density of the international rice trade network and the international wheat trade network decreased significantly after the first quarter of 2022 (see Figure 2f). It would reflect the impact of the Russia-Ukraine conflict on the international rice and wheat trade. Figure 2g presents that the properties of bidirectional trading relationships between economies in the international crop trade networks were stable after 2016 but showed a decreasing trend after the first quarter of 2022. From Figure 2h, we can see that the efficiency of the international crop trade networks decreased after the conflict. It implies that there are less efficient pathways for propagating crop trade flows. Furthermore, natural connectivity is stable in general.

**Figure 3:**
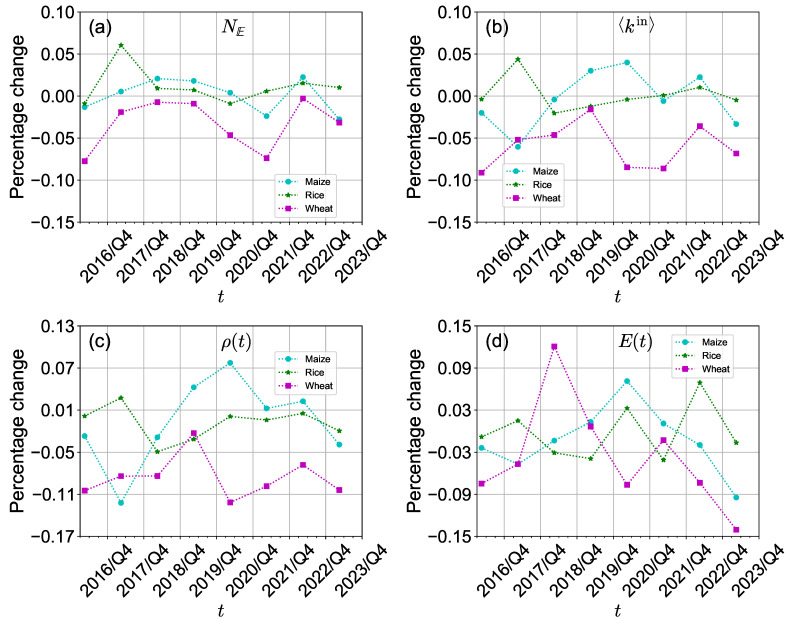
(**a**–**d**) Percentage change in the structure of the three international crop trade networks comparing two adjacent quarters (the first and second quarter) from 2016 to 2023.

The Russia-Ukraine conflict has had a more significant impact on the structure of the international wheat trade network than on the international maize trade network and international rice trade network in the short term. Figure 3 provides an overview of the percentage change in the number of edges, average in-degree, density, and efficiency between the first and second quarters from 2016 to 2023. In 2022/Q2, the percentage changes in the number of edges and average in-degree of the international maize trade network and the international rice trade network were both positive (see Figure 3a,b). This indicates that the number of edges and average in-degree of the international maize and rice trade networks kept increasing. Although these networks witnessed more substantial decreases in these two metrics before 2022, it remains inconclusive whether these changes were directly influenced by the Russia-Ukraine conflict. However, the international wheat trade network exhibited a noteworthy decline in average in-degree and density specifically during 2022/Q2 compared to 2022/Q1, suggesting a negative impact on international wheat trade stemming from the conflict. Furthermore, the density of the international wheat trade network experienced significant decreases in 2018/Q2 and 2022/Q2 (see Figure 3c). This indicates that the connectivity of the international wheat trade network decreased. The reason for the 2018 decrease is that Northern and Eastern Europe experienced wheat yield losses due to extreme weather [61]. Meanwhile, the conflict has disrupted the structure of the international wheat trade network.

**Figure 4:**
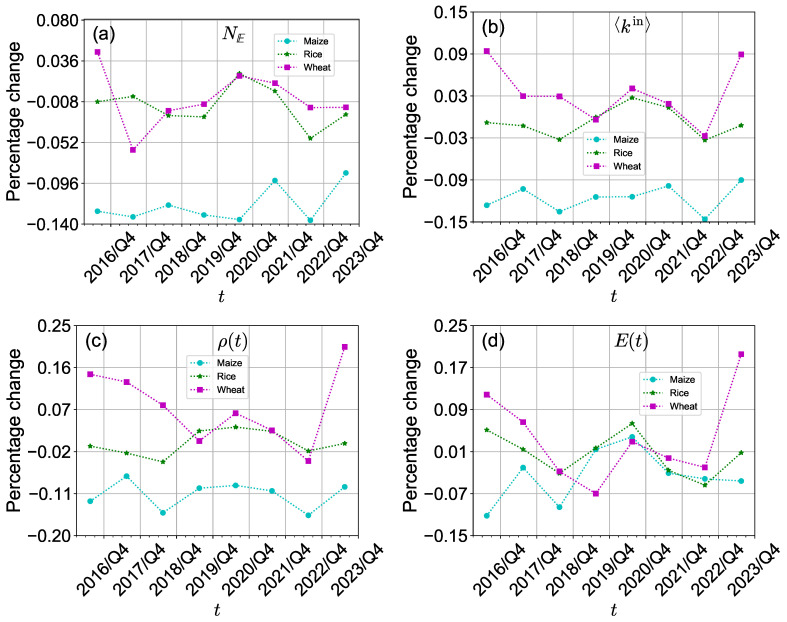
(**a**–**d**) Percentage change in the structure of the three international crop trade networks comparing two adjacent quarters (the second and third quarter) from 2016 to 2023.

All three international crop trade networks experienced disturbances within six months following the conflict. As shown in Figure 4, the percentage changes in the number of edges, average in-degree, density, and efficiency of the three international crop trade networks were all negative. This indicates that the structure of the international crop trade networks was disrupted within a period of time after the Russia-Ukraine conflict. Interestingly, the number of edges and density increased in 2023/Q3 instead of decreasing. Notably, the international wheat trade network experienced a significant rise in average in-degree, density, and efficiency during this quarter. Conversely, the number of edges of the international crop trade networks exhibited a decrease. These findings suggest that while the conflict influenced the structure and connectivity of the international crop trade networks, it did not impact the participation of economies in international crop trade.

**Figure 5:**
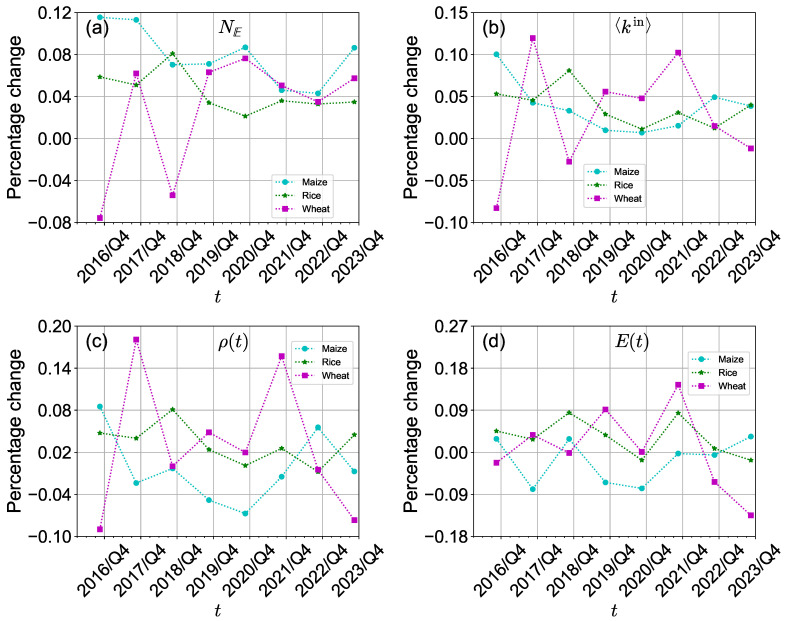
(**a**–**d**) Percentage change in the structure of the three international crop trade networks comparing two adjacent quarters (the third and fourth quarters) from 2016 to 2023.

The ongoing Russia-Ukraine conflict has consistently exerted a negative impact on international crop trade networks, particularly on the international wheat trade network. As depicted in Figure 5a,b, the percentage changes in the number of edges and average in-degree of the international crop trade networks were positive but decreased in 2022/Q4. This indicates that although the number of edges and average in-degree of the international crop trade networks increased, the rate of increase diminished, likely due to the impact of the Russia-Ukraine conflict. In 2023/Q4, the number of edges increased, while the average in-degree of the international wheat trade network declined. As shown in Figure 5c,d, the density and efficiency of the international wheat trade network exhibited a more substantial reduction in 2022/Q4 and 2023/Q4. This can be partly attributed to the escalating conflict, which has hindered the development of free trade.

Overall, the impact of the Russia-Ukraine conflict on the international crop trade networks differs across different crop sectors. The international wheat trade network is shown to face the most severe disturbance. Wheat yield losses in Ukraine and wheat export restrictions would explain the shifts in the structure of the international wheat trade network. Russia and Ukraine are major wheat producers and traders, especially for European economies. Both Russia and Ukraine have imposed sanctions on each other, including trade restrictions. These measures have hindered the flow of wheat and other agricultural products between the economies and affected the availability of wheat in European markets.

### 3.2. Impact on the Aggregate International Food Trade Network

**Figure 6:**
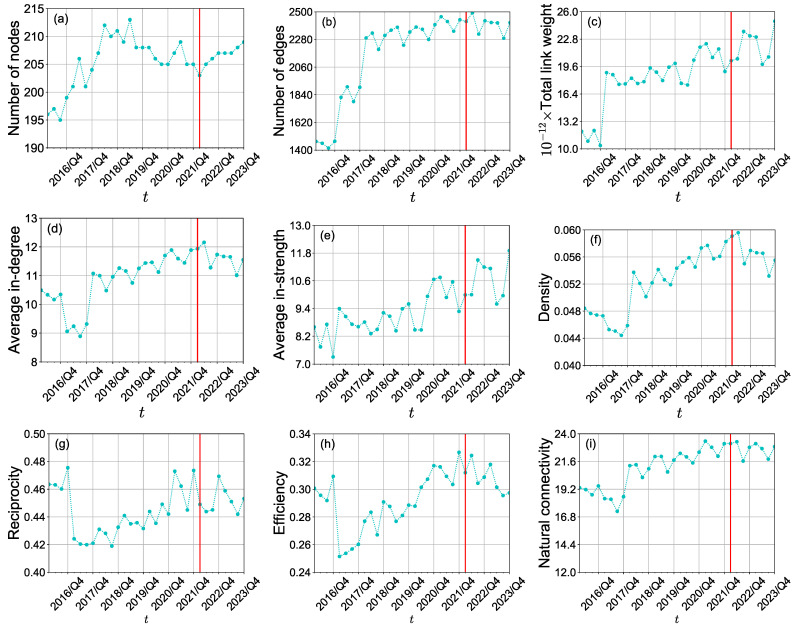
(**a**–**i**) Quarterly evolution of the structure of the aggregate international food trade network from 2016/Q1 to 2023/Q4.

We construct the aggregate international food trade network by aggregating maize, rice, and wheat to gain some insight into the impact of the Russia-Ukraine conflict on the total crop trade. The number of economies included in the aggregate international food trade network was not affected by the conflict. We show the quarterly evolution of the structure of the aggregate international food trade network from 2016/Q1 to 2023/Q4 in Figure 6. We find that all topological metrics display an overall upward trend, which differs from the behavior observed in the individual international crop trade networks. Notably, the number of nodes, the link weights, the average in-strength, and the link reciprocity maintained a consistent trend in 2022. Nevertheless, changes were observed in the number of edges, average in-degree, density, efficiency, and natural connectivity following the onset of the conflict.

**Figure 7:**
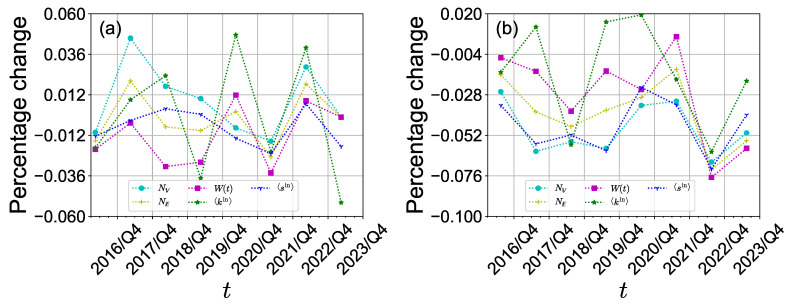
Percentage change r(t) in the structure of the aggregate international food trade network comparing the first quarter with the second quarter (**a**) and the second quarter with the third quarter (**b**) from 2016 to 2022.

Our focus is solely on analyzing the structural changes in the aggregate international food trade network during the ongoing conflict. Figure 7a,b illustrates the percentage change in the aggregate international food trade network’s structure, comparing the first quarter to the second quarter and the second quarter to the third quarter, respectively, from 2016 to 2022. Due to the absence of significant structural changes between the third and fourth quarters, we omit these results. It is evident that in 2019/Q2, there was a significant decrease in the number of edges, average in-degree, density, efficiency, and natural connectivity. Another notable finding is that these network metrics increased in 2022/Q2 but decreased in 2023/Q2 as shown in Figure 7a. Additionally, Figure 7b shows that these network metrics continued to decline in 2023/Q3. This suggests that the conflict initially had a minimal impact on the aggregate international food trade network within a short timeframe but eventually had a negative effect as the conflict endured. This finding contrasts somewhat with the results of the international wheat trade network, which show a decrease in connectivity. One possible explanation for this disparity lies in the substitution effect among maize, rice, and wheat [11]. Since these staple crops can often be used as substitutes for one another in various food products, when the production or exports of wheat decline, consumers and businesses tend to shift their preferences to alternative crops like rice and maize. This increased demand for rice and maize can lead to higher exports of these crops to meet international market demand. Consequently, the aggregate international food trade network was not severely affected in the short term.

### 3.3. Impact on Economies

To gain a deeper understanding of the Russia-Ukraine conflict’s impact across economies, we conduct economy-scale analyses from two aspects. These include the topological properties of Russia and Ukraine and the trade relationships between NATO economies and both Russia and Ukraine. Russia and Ukraine present different responses to the conflict.

**Figure 8:**
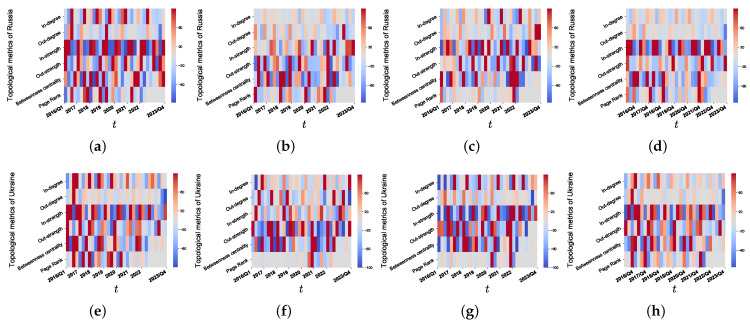
(**a**–**h**) Quarterly percentage change in in-/out-degrees, in-/out-strengths, betweenness centrality, and PageRank for Russia and Ukraine. The columns from left to right, respectively, describe maize, rice, soybean, and wheat.

Figure 8 presents the quarterly percentage change in in-/out-degrees, in-/out-strengths, betweenness centrality, and PageRank for Russia and Ukraine. As shown in Figure 8a–d, the topological properties except in-strength of Russia decreased in 2022/Q2. However, a partial recovery is observed in 2023/Q4, where some of these properties show an increase. These findings suggest that the Russia-Ukraine conflict has had a negative short-term impact on Russia’s crop trade. Compared to Russia, Ukraine is less affected by the Russia-Ukraine conflict, as shown in Figure 8e–h.

The relationships between NATO economies and both Russia and Ukraine are complicated. As a political and military alliance, NATO has consistently shown support for Ukraine and expressed criticism of Russia’s actions. NATO has strongly condemned Russia’s annexation of Crimea and its involvement in the conflict in Eastern Ukraine. Consequently, this has resulted in a deterioration of relations between NATO and Russia, with significant implications for trade and cooperation. Thus, our analysis also reveals heterogeneity in trade structure between Russia and Ukraine.

**Figure 9:**
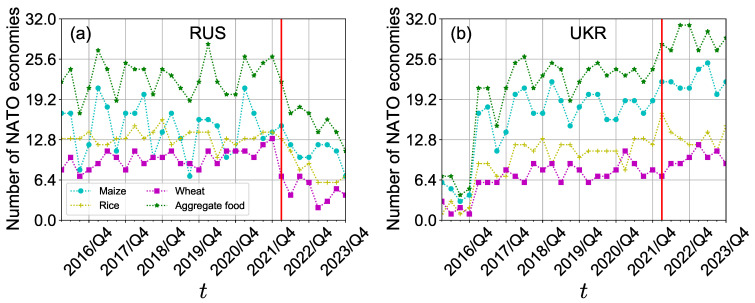
The number of NATO economies importing or exporting crops from or to Russia (**a**) and Ukraine (**b**) from 2016/Q1 to 2023/Q4.

Figure 9 presents the number of NATO economies that have trade links with Russia and Ukraine from 2016/Q1 to 2023/Q4. Notably, after the conflict began, there was a significant decline in trade links between Russia and NATO economies for all crops and aggregate food, as depicted in Figure 9a. This decline can be attributed to the conflict’s containment effect on trade relationships between Russia and NATO economies. However, the impact of the conflict eased over time. Moreover, Figure 9b shows that Ukraine increased its trade links with NATO economies for all crops after 2016, and while the trade links for maize and rice showed a slight decrease following the onset of the conflict, they eventually stabilized, alleviating the trade relationship strain. Conversely, trade links for wheat and aggregate food exhibited a slight increase after 2022/Q1 and 2022/Q4. This suggests that the conflict had a more severe impact on the crop trade relationships between Russia and NATO economies compared to those between Ukraine and NATO economies.

**Figure 10:**
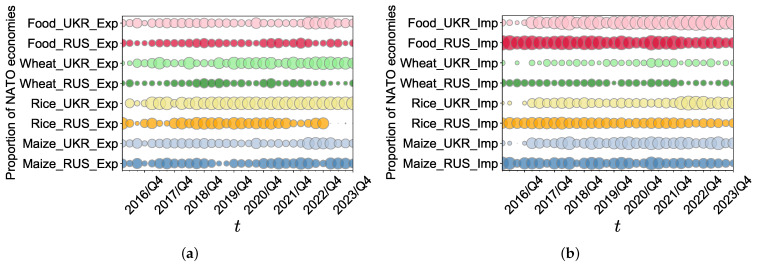
(**a**,**b**) Evolution of the proportion of food caloric exporting volumes and importing volumes between Russia and NATO economies to all 24 economies, and between Ukraine and NATO economies to all 24 economies from 2016/Q1 to 2023/Q4.

We display the evolution of the proportion of food caloric trade volumes between Russia and NATO economies and between Ukraine and NATO economies to all 24 economies from 2016/Q1 to 2023/Q4 in Figure 10. We find that following the occurrence of the conflict, the proportion of maize and rice exports between Russia and NATO economies, as well as between Ukraine and NATO economies, did not undergo significant changes. This indicates that these crop exports remained relatively stable amidst the conflict. However, a substantial decline in the proportion of wheat and aggregate food exports between Russia and NATO economies is evident. This decline suggests a noticeable impact of the conflict on these specific trade relationships, with a decrease in the relative volume of these exports. Furthermore, the proportion of NATO economies’ imports from Russia and Ukraine for the three crops, as well as for overall food, from 2016/Q1 to 2023/Q4, remained unaffected by the conflict.

**Figure 11:**
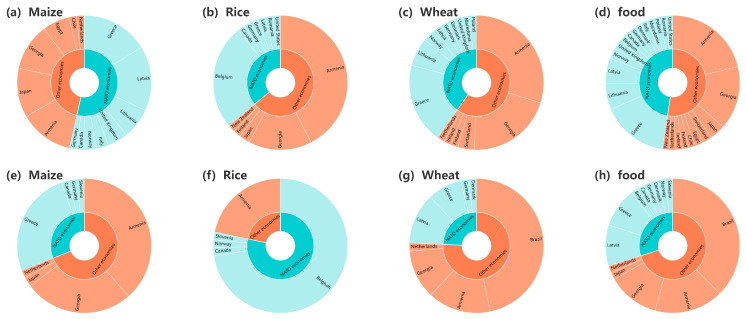
Russia’s crop export structure in 2021/Q4 (**a**–**d**) and 2022/Q4 (**e**–**h**). The inner circle shows the ratio of crop calories imported from Russia by NATO economies and other economies, with blue indicating NATO economies and orange indicating other economies. The outer circle displays the ratio of crop calories imported from Russia by different economies.

**Figure 12:**
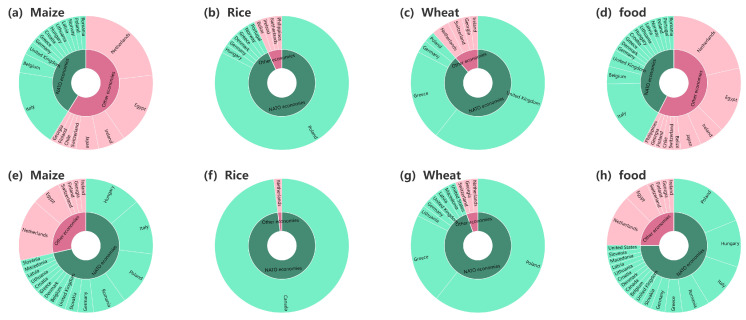
Ukraine’s crop export structure in 2021/Q4 (**a**–**d**) and 2022/Q4 (**e**–**h**). The inner circle shows the ratio of crop calories imported from Ukraine by NATO economies and other economies, with green indicating NATO economies and pink indicating other economies. The outer circle displays the ratio of crop calories imported from Ukraine by different economies.

Since Russia and Ukraine are major crop exporters, we compare Russia’s and Ukraine’s crop export structures in 2021/Q4 and 2023/Q4 in Figure 11 and Figure 12. It is worth noting that the cessation of export trade between Russia and some NATO economies resulted in a marked decline in the proportion of maize, wheat, and aggregate food exports to NATO economies to Russia’s total maize, wheat, and aggregate food exports after the Russo-Ukrainian conflict, as shown in Figure 11a,c–e,g,h. Conversely, as illustrated in Figure 11b,c, there was a discernible increase in the proportion of rice exports to NATO economies to Russia’s total rice exports. In contrast to Russia, Ukraine experienced a different scenario, as demonstrated by Figure 12, which showcases an escalation in maize, wheat, and aggregate food export trade with NATO economies, leading to an increase in the proportion of exports to NATO economies to Ukraine’s total maize, rice, wheat, and aggregate food exports.

Overall, the impact of the Russia-Ukraine conflict differs across economies and crops. The conflict has different impacts on Russia and Ukraine. It significantly altered the topological properties of Russia and had a notable impact on the trade relationships between Russia and NATO economies, particularly regarding wheat. Although less so than in Russia, the conflict also had an impact on Ukraine. The findings indicate that the conflict had a more substantial impact on Russia’s trade dynamics and network properties than on Ukraine.

## 4. Discussion

The Russia-Ukraine conflict has sparked global apprehension regarding international trade and food security. As significant grain exporters, both Russia and Ukraine play a crucial role, accounting for approximately 40% of global grain exports. Notably, these economies were responsible for about 30% of the world’s wheat exports in 2021. Consequently, the conflict has triggered declines in the global food supply and substantial increases in global food prices. In this paper, we consolidate three international crop trade networks and an aggregate international food trade network and compare structural changes by using crop trade data from the UN Comtrade Database. Additionally, we analyze the topological attributes of Russia and Ukraine and the shifts in crop trade relationships between NATO economies and both Russia and Ukraine. Through this comprehensive analysis, we aim to provide valuable insights into the impact of the conflict on the network structures and trade dynamics within the global agrifood sector.

First, we present the structural evolution of three international crop trade networks and the aggregate international food trade network over the period from the first quarter of 2016 to the fourth quarter of 2023, including the number of nodes, edges, total edge weight, average degree, average strength, density, clustering coefficient, efficiency, and natural connectivity. We found that the Russia-Ukraine conflict has affected the structure of the international crop trade networks and the aggregate international food trade network. Furthermore, the impact of the Russia-Ukraine conflict on the international crop trade networks differs across different crop sectors. It seems plausible to assume that wheat yield losses in Ukraine and wheat export restrictions make the international wheat trade network face the most severe disturbance.

In terms of the aggregate international food trade network, all topological metrics display an overall upward trend, which differs from the behavior observed in the individual international crop trade network. Notably, the number of nodes, the link weights, the average in-strength, and the link reciprocity maintained a consistent trend in 2022. Nevertheless, changes were observed in the number of edges, average in-degree, density, efficiency, and natural connectivity following the onset of the conflict. Another notable finding is that these network metrics increased in 2022/Q2 but decreased in 2023/Q3. This suggests that the conflict initially had a minimal impact on the aggregate international food trade network within a short time but eventually had a negative effect as the conflict endured. This finding contrasts somewhat with the results of the international wheat trade network, which show a decrease in connectivity. One possible explanation for this disparity lies in the substitution effect among maize, rice, and wheat [11].

We examine the effects of the Russia-Ukraine conflict specifically on Russia and Ukraine. Our study investigates the quarterly percentage changes in in-/out-degrees, in-/out-strengths, betweenness centrality, and PageRank for both countries. The findings indicate that the Russia-Ukraine conflict has had a negative short-term impact on Russia’s crop trade. In comparison, Ukraine has experienced relatively lesser effects from the conflict. Additionally, we analyze the trade relationships between NATO economies and both Russia and Ukraine before and after the conflict. It is observed that the conflict has had a more significant impact on the crop trade relationships between Russia and NATO economies, compared to Ukraine and NATO economies. The discontinuation of export trade between Russia and certain NATO economies has resulted in a notable decline in the proportion of maize, wheat, and aggregate food exports from Russia to NATO economies in relation to its total maize, wheat, and aggregate food exports.

Our study extends the existing literature by providing a detailed analysis of the structural changes within international crop trade networks and the aggregate international food trade network in response to the Russia-Ukraine conflict, and while prior studies have broadly examined geopolitical impacts on global food security and trade dynamics [41,42,44], our focus on network metrics such as the number of nodes, the number of edges, link weights, average in-degree, average in-strength, density, reciprocity, efficiency, and natural connectivity, offers a novel perspective. For instance, while Liverpool-Tasie et al. [11] discuss substitution effects among staple crops, our analysis highlights specific disruptions in the international wheat trade network due to export restrictions and yield losses in Ukraine.

In contrast to previous economic analyses, which often overlook network intricacies [51], our findings underscore the asymmetrical impacts on Russia and Ukraine, revealing distinct vulnerabilities and adaptation strategies within these agricultural networks. This nuanced approach enhances our understanding of how geopolitical conflicts can reshape global food trade dynamics and market stability, aligning with studies emphasizing resilience and adaptive capacity in food supply chains.

Several themes and limitations emerge from our analysis that shape the implications of our findings. Firstly, while our study provides insights into immediate impacts on crop trade networks, longitudinal assessments are crucial to evaluating long-term resilience and adaptive strategies amid ongoing geopolitical uncertainties. Secondly, reliance on UN Comtrade data limits our ability to capture informal trade channels and regional nuances in agricultural trade dynamics. Future research should incorporate more granular data sources and qualitative assessments to enhance the robustness of our findings. Thirdly, the observed decline in connectivity and trade volumes within the international wheat trade network underscores vulnerabilities in global food supply chains. Addressing these vulnerabilities requires policy interventions that promote diversified sourcing strategies and strengthen regional food security frameworks. Lastly, our study highlights implications for achieving Sustainable Development Goals (SDGs), particularly SDG 2 (Zero Hunger), and SDG 17 (Partnerships for the Goals). The disruption caused by the conflict underscores the need for international cooperation and adaptive governance mechanisms to ensure food security and mitigate socio-economic impacts on vulnerable populations.

## 5. Conclusions

The Russia-Ukraine conflict has significantly impacted international trade networks, particularly within the global agrifood sector. Our comprehensive analysis reveals that the conflict has disrupted the structural integrity of international crop trade networks, with notable effects on wheat trade. Despite an initial increase in network metrics, such as the number of edges and average in-degree in early 2022, these metrics eventually declined, reflecting the broader impacts of the conflict on trade efficiency and connectivity.

Interestingly, while the international wheat trade network experienced marked disruptions, the aggregate international food trade network exhibited a more resilient structure. This resilience is likely due to the substitution effect among staple crops like maize, rice, and wheat, allowing the global food supply chain to adapt despite the challenges posed by the conflict. Furthermore, our study highlights the differential impacts on trade relationships between NATO economies and both Russia and Ukraine. Post-conflict, trade links between Russia and NATO economies declined significantly, particularly for wheat and aggregate food, whereas Ukraine managed to increase its trade links with NATO economies. This divergence underscores the varying geopolitical and economic dynamics at play. The observed changes in trade patterns underscore the complex interplay between geopolitical events and global food security. The conflict has not only affected trade structures but has also led to strategic shifts in crop export relationships, particularly between NATO economies and the conflicted nations. These findings provide valuable insights into the adaptive responses within international trade networks during periods of geopolitical instability, emphasizing the need for robust and diversified trade strategies to mitigate such disruptions.

In summary, the Russia-Ukraine conflict has had profound short-term and potentially long-term effects on international crop trade networks. Continued monitoring and analysis are essential to understanding the evolving dynamics and to developing strategies that enhance the resilience of global food trade systems against geopolitical disruptions. Our analysis offers critical insights into the structural dynamics of international crop trade networks amidst the Russia-Ukraine conflict. However, interdisciplinary research is essential to comprehensively address the complex challenges facing global food security in the 21st century.

## Figures and Tables

**Table 1:** Economies that report crop trade datasets during the period from 2016/1 to 2023/12. Economies with superscript * belong to the North Atlantic Treaty Organization (NATO). We also show the regions to which the reporting economies belong.

Region	Maize	Rice	Wheat
Africa	Egypt	Egypt	
		Mauritius	
	Rwanda	Rwanda	
		São Tomé and Principe	
Asia	Armenia	Armenia	Armenia
	Georgia	Georgia	Georgia
	Japan	Japan	Japan
	Philippines	Philippines	
Europe	Belgium *	Belgium *	
	Bosnia and Herz.	Bosnia and Herz.	Bosnia and Herz.
	Switzerland	Switzerland	Switzerland
	Germany *	Germany *	Germany *
	Denmark *	Denmark *	Denmark *
	Finland	Finland	Finland
	United Kingdom *		United Kingdom *
	Greece *	Greece *	Greece *
	Croatia *	Croatia *	
	Hungary *	Hungary *	
	Ireland	Ireland	Ireland
	Iceland *		
	Italy *	Italy *	
	Latvia *	Latvia *	Latvia *
	Lithuania *	Lithuania *	Lithuania *
	Macedonia *	Macedonia *	Macedonia *
	The Netherlands	The Netherlands	The Netherlands
	Norway *	Norway *	Norway *
	Poland *	Poland *	Poland *
		Portugal *	
	Romania *	Romania *	
	Slovakia *		
	Slovenia *	Slovenia *	
	Sweden	Sweden	
North America	Belize	Belize	
	Barbados	Barbados	
	Canada *	Canada *	Canada *
	Grenada	Grenada	
	Mexico	Mexico	
	El Salvador	El Salvador	El Salvador
	United States *	United States *	United States *
Oceania	Australia	Australia	Australia
		Fiji	
	New Zealand	New Zealand	New Zealand
South America	Bolivia	Bolivia	
	Brazil	Brazil	Brazil
	Chile	Chile	Chile

## Data Availability

The original data presented in the study are openly available in https://comtradeplus.un.org.
